# Alzheimer's Disease: The Role of Microglia in Brain Homeostasis and Proteopathy

**DOI:** 10.3389/fnins.2017.00680

**Published:** 2017-12-12

**Authors:** Kevin A. Clayton, Alicia A. Van Enoo, Tsuneya Ikezu

**Affiliations:** ^1^Department of Pharmacology and Experimental Therapeutics, Medical School, Boston University, Boston, MA, United States; ^2^Department of Neurology, Medical School, Boston University, Boston, MA, United States

**Keywords:** Alzheimer's disease, microglia, neurodegeneration, amyloid-beta peptide, tau protein, neuroinflammation, proteopathy

## Abstract

Brain aging is central to late-onset Alzheimer's disease (LOAD), although the mechanisms by which it occurs at protein or cellular levels are not fully understood. Alzheimer's disease is the most common proteopathy and is characterized by two unique pathologies: senile plaques and neurofibrillary tangles, the former accumulating earlier than the latter. Aging alters the proteostasis of amyloid-β peptides and microtubule-associated protein tau, which are regulated in both autonomous and non-autonomous manners. Microglia, the resident phagocytes of the central nervous system, play a major role in the non-autonomous clearance of protein aggregates. Their function is significantly altered by aging and neurodegeneration. This is genetically supported by the association of microglia-specific genes, *TREM2* and *CD33*, and late onset Alzheimer's disease. Here, we propose that the functional characterization of microglia, and their contribution to proteopathy, will lead to a new therapeutic direction in Alzheimer's disease research.

## Introduction

Aging results in a loss of proteostasis that is characteristic of many neurodegenerative disorders. During aging, the mechanisms responsible for protein synthesis, post-translational modifications, and clearance, cumulatively known as “proteostasis,” become dysregulated in the central nervous system (CNS). Impairments in proteostasis result in the accumulation of misfolded proteins as intracellular aggregates, such as neurofibrillary tangles and Lewy bodies, or extracellular plaques, such as senile and prion plaques, which ultimately lead to conditions termed “proteopathies.” Alzheimer's disease (AD) is diagnosed with the development of two unique pathologies, senile plaques and neurofibrillary tangles. A comprehensive understanding of the brain cells responsible for the clearance of protein aggregation due to dysfunction is critical to fully elucidate the etiology of AD. This mechanism is largely understood to be a cell autonomous process, namely executed by neurons. However, non-autonomous processes that may also be involved have recently become the subject of extensive investigation. One representative cell type controlling brain proteostasis is microglia. Interestingly, their functions are largely affected by aging. This is genetically supported by the significant genome-wide association of microglia-specific *TREM2* gene with LOAD (Guerreiro et al., 2013; Jonsson et al., 2013). Microglia are the resident phagocytes of the CNS and are implicated in the pathogenesis of many neurocognitive disorders, including neurodevelopmental and neurodegenerative diseases. They are implicated in the phagocytosis and degradation of pathological protein aggregates. Numerous studies have been published recently depicting the changes that cause microglia to become dysfunctional during aging and disease. Once microglia become dysfunctional, they further contribute to CNS destabilization in response to protein aggregates, which ultimately leads to brain degeneration. One prominent aspect of microglial dysfunction is their role in chronic neuroinflammation, a phenomenon in which immune cells recognize and pervade the ailing tissue causing damage through both antigen-specific and non-specific mechanisms. Several groups have recently provided a comprehensive characterization of microglial phenotype in order to elucidate the mechanisms by which microglial dysfunction disrupts the CNS microenvironment (Matcovitch-Natan et al., 2016; Keren-Shaul et al., 2017; Krasemann et al., 2017).

These new concepts considering the causal relationships between microglial dysfunction, neuroinflammation, and protein aging, introduce the question of how novel therapies may halt or reverse the contribution of microglia to the spread of proteopathy and neurodegeneration. Here, we summarize clinical and preclinical studies aimed to prevent protein aggregation or restore homeostatic microglial function. Furthermore, we overview the physiological function of microglia, their changes in response to aging, and their specific neurodegenerative phenotype leading to proteopathy in AD.

### Aging and proteopathies

Most neurodegenerative disorders are pathologically characterized as proteopathies (Walker and LeVine, 2000). Aging can impact multiple aspects of proteostasis: production, folding, posttranslational modification, and clearance in several pathways, including secretion and autophagosomal, endolysosomal, and proteasomal degradation (Kaushik and Cuervo, 2015), all of which are demonstrated to affect protein aggregation when impaired. Aggregated proteins, such as amyloid-beta peptides (Aβ), are inherently cytotoxic *in vitro*, causing stress and stimulating synaptic loss, mitochondrial dysfunction, and eventually apoptosis in neurons (Sakono and Zako, 2010). We will mainly describe the metabolism of Aβ and tau in this review.

The production of Aβ from amyloid precursor protein (APP) is the most well-studied component of AD development, especially in early-onset Alzheimer's disease (EOAD) (Rovelet-Lecrux et al., 2006; Sleegers et al., 2006; McNaughton et al., 2012). In addition to the well-known mutations proximal to the α, β, and γ-processing of APP, several mutations in the promoter region of *APP* have been reported and contribute to enhanced *APP* gene expression (Athan et al., 2002; Theuns et al., 2006; Hooli et al., 2012; Rodgers et al., 2012). However, the majority of sporadic cases do not show increased expression of APP by aging. Rather, there is a reduction in the amount of Aβ in the cerebrospinal fluid (CSF), suggesting its absorption into amyloid plaques, rather than export to the periphery. Researchers have increasingly argued that sporadic or late-onset AD is more likely caused by a reduction in clearance of Aβ than by its overproduction (Mawuenyega et al., 2010).

There is also a possibility for an age-related shift in APP processing toward the amyloidogenic rather than non-amyloidogenic pathway. This results in the production of pathogenic Aβ, as opposed to the non-pathogenic p3 peptide. One study reported that age did not have an effect on γ-secretase production of Aβ42 (Dewachter et al., 2000). However, the expression of beta-site APP converting enzyme 1 (BACE1), the enzyme designated as β-secretase, is elevated in the AD brain (Li et al., 2004; Zhao et al., 2007). Furthermore, primary cortical neurons were shown to up-regulate *BACE1* expression in response to Aβ42 exposure (Mamada et al., 2015). This suggests that Aβ42 production may be self-perpetuating via up-regulation of BACE1 expression in neurons. Given that BACE1 acts on APP primarily in endosomal compartments, age-related increases in early endosome volume could result in increased BACE1 processing of APP (Cataldo et al., 2000). Another possibility is the reduced activity or expression of α-secretase, ADAM10, a component of the non-amyloidogenic pathway. Reduced non-amyloidogenic processing of APP was reported to occur as a result of cellular aging (Kern et al., 2006), although overall activity and expression was increased in cognitively normal subjects (Schuck et al., 2016). This suggests that an age-dependent reduction of ADAM10 function is specific to AD subjects.

Reduced clearance of Aβ has emerged as the central mechanism of amyloid plaque formation in AD. Aβ is thought to be cleared via interstitial fluid (ISF) drainage into the blood vessels, and brought across the blood brain barrier (BBB) into the peripheral bloodstream. There are mutations in APP reported in AD cases that appear to reduce transport of Aβ from the CSF into the blood (Monro et al., 2002). Receptor for advanced glycation end products (RAGE) is proposed to be expressed in the BBB and responsible for shuttling Aβ from the bloodstream into the brain, while LRP1 is responsible for efflux of Aβ42 out of the brain (Deane et al., 2009). However, LRP1 expression in the BBB is reduced in rodents, primates, and humans (Shibata et al., 2000; Deane et al., 2004; Zerbinatti et al., 2004; Donahue et al., 2006). For this reason, RAGE antagonists, which have already been proposed to have effective anti-inflammatory properties, are being further explored for clinical development (see Table 1). In addition to the transport of Aβ out of the ISF to the blood, the CNS is equipped with several other clearance mechanisms, principally mediated via Aβ degrading enzymes. These include enzymes derived from the M13 zinc-binding membrane metalloendopeptidase (such as insulin degrading enzyme), Type II integral membrane bound glycoproteins (NEP-2, ECE-1), membrane-bound zinc metalloproteinase (such as matrix metalloproteinase 2 and 9), thiol-metalloendopeptidase, matrix metalloproteinase, and members of the serine and cysteine protease families (see Miners et al., 2011 for a complete list). One well-investigated protease, neprilysin (NEP), appears to be critical in this pathway, demonstrated by the effectiveness of a combination of NEP inhibitors, over-expression of *NEP*, and genetic disruption of *NEP* in increasing proteolytic cleavage and clearance of Aβ (Marr et al., 2003; Dolev and Michaelson, 2004; Nisemblat et al., 2008; Hafez et al., 2011; Takamatsu et al., 2014). Aβ clearance also occurs extracellularly, via microglia-mediated phagocytosis. The effect of aging on this process is further discussed in following sections.

**Table 1:** A comprehensive list of all of the drugs in clinical development that aim to reduce either Aβ pathology, tau pathology, or inflammation within the past five years categorized by treatment strategy and drug class.

**Category**	**Drug class**	**Mechanism**	**Compound name**	**Current status**
Inflammation	Anti-inflammatories (Ferretti et al., 2012; Tawakol et al., 2014; Hori et al., 2015)	Anti-inflammatories act through a variety of interactions.	Cromoglicic acid	Approved (alternate indication)
			ALZT-OP1	Approved (alternate indication)
			CHF 5074	Phase 2
	RAGE Antagonists (Srikanth et al., 2011; Gilham et al., 2016)	RAGE increases pathogenic pro-inflammatory signaling in diabetes, AD, and cancer. Antagonists may alleviate the deleterious effects, but may also reduce amyloid deposition.	Rilapladib ALZT-OP1	Phase 2 Phase 3
			GSK2647544	Phase 1
			Minocycline	Approved (alternate indication)
			Azeliragon	Phase 3
	Tumor necrosis factor alpha inhibitor (Butchart et al., 2015; Gilham et al., 2016)	Traditionally a cancer drug, Etanercept may lower the effects of heightened levels of tumor necrosis factor alpha and deleterious inflammation.	Etanercept	Approved (alternate indication)
Tau-related pathology	Microtubule Stabilizers (Mitchell et al., 2016)	Dysfunction of phosphorylated tau results in impaired microtubule stabilizing function, synaptic shrinkage, and eventually neuronal death. Microtubule-stabilizing agents may alleviate the deficit caused by tau phosphorylation.	TPI-287	Phase 1
	Inhibitors of Tau Aggregation (Harrington et al., 2015)	Small molecules that bind pathological versions of tau and prevent aggregation may help reduce overall toxicity.	TRx0237	Phase 3
	Vaccine against Tau (Theunis et al., 2013; Kontsekova et al., 2014)	Use second-generation immunotherapy to stimulate the brains innate immune system to increase neurofibrillary tangle clearance.	AADvac1 ACI-35	Phase 2 Phase 1
	Src/abl family of kinases inhibitor (Nygaard et al., 2014)	Fyn phosphorylates tau after exposure to pathogenic Aβ. Therefore, inhibitors may prevent or reduce the generation of pathological tau.	Saracatinib	Phase 2
	Antibodies against Tau	Antibodies against pathogenic Tau seek to utilize the brain's natural immune system to clear Aβ faster.	ABBV-8E12	Phase 2
			RO7105705	Phase 1
Reduction in Aβ	BACE Inhibitors (He et al., 2013; Eketjäll et al., 2016)	Inhibits the action of pathogenic β-secretase cleavage of APP, reducing overall amyloid burden.	AZD3293	Phase 3
			JNJ-54861911	Phase 2/3
			LY3202626	Phase 2
			CNP520	Phase 2/3
			E2609	Phase 3
	Antibodies against Aβ (Bard et al., 2000; Dodel et al., 2013)	Antibodies against pathogenic Aβ seek to utilize the brain's natural immune system to clear Aβ faster.	BAN2401	Phase 2
			Gantenerumab	Phase 3
			GSK933776	Phase 2
			LY3002813	Phase 1
			LY3303560	Phase 1
			MEDI1814	Phase 1
			SAR228810	Phase 1
			AAB-003	Phase 1
			Aducanumab	Phase 3
			Crenezumab	Phase 3
			Gamunex	Approved (alternate indication)
			KHK6640	Phase 1
	Small Molecule Aβ inhibitors (McLaurin et al., 2006; Habchi et al., 2016)	Small molecules are believed to either bind Aβ42 and Aβ40 peptides early, preventing nucleation, or inhibiting organization of higher-level tertiary and quaternary structures.	ELND005	Phase 2
	RXR-selective analogues (Tai et al., 2014)	These receptors ameliorate loss-of-function associated with ApoE, decreasing Aβ burden and improving synaptic viability.	Bexarotene	Approved (alternate indication)
	Phosphodiesterase 9 Inhibitors (Su et al., 2016)	PDE9 inhibitors halt Aβ aggregation, thus reducing abundance and associated toxicity of senile plaques.	BI 409306 BPN14770	Phase 2 Phase 1
	Beta amyloid vaccines (Wiessner et al., 2011)	Using second-generation immunotherapy to stimulate the brain's innate immune system to increase Aβ clearance.	CAD106	Phase 2/3
			MER5101	Phase 1
			UB-311	Phase 2
			ACI-24	Phase 1/2
	Purinoceptor P2Y6 agonists (Koizumi et al., 2007)	Stimulation of the P2Y6 receptor increases microglia phagocytosis and associated clearance of Aβ.	GC021109	Phase 1
	Gamma Secretase Modulators (Imbimbo et al., 2009; Imbimbo and Giardina, 2011)	Modulate γ-secretase to process pathological Aβ42 more readily into non-toxic forms.	NGP 555 CHF 5074 EVP-0962	Phase 1 Phase 2 Phase 2
	Inhibitors of Aβ synthesis (Maccecchini et al., 2012)	Binding of APP mRNA prevents translation, thus reducing amyloid burden in the subject.	Posiphen	Phase 1/2
	Sigma 2 receptor ligands (Izzo et al., 2014)	These ligands bind to the sigma 2 receptor, inhibiting binding of Aβ fragments and associated synaptic toxicity.	CT1812	Phase 1/2
	Glutaminyl cyclase inhibitors (Morawski et al., 2014)	Glutaminyl cyclase is a metalloenzyme that catalyzes the cyclization of pathogenic Aβ, forming pGlu-Ab, which is a highly toxic constituent of senile plaques.	PQ912	Phase 2
	Dihydropyridine calcium channel blocker	Serves as an anti-hypertensive with Aβ deposition prevention properties (Paris, 2010).	Nilvadine	Phase 3
	SNRI (Chalermpalanupap et al., 2013)	Reducing NET activity has the potential to reduce amyloid burden.	Atomoxetine	Phase 2

*Drugs in green are already approved by the FDA for a condition separate from AD. Drugs in red have been granted Fast Track privileges for AD clinical development*.

### Microglial origin and presence in the CNS

This first reports of cells exhibiting microglia-like phenotypes came from the work of Nissl and Robertson in the late nineteenth century (Gomez-Nicola and Perry, 2015). It wasn't until the late 1930's that these cells were differentiated from other glial cells and received the name of “microglia” by Pio del Rio-Hortega. Hortega used silver staining techniques to describe microglial morphology and introduced the idea of microglia as ramified resting cells (Ginhoux et al., 2013). He pioneered the idea that microglia have the ability to change morphology, migrate, and proliferate in response to their microenvironment and described their basic functional roles as phagocytic cells (Ginhoux et al., 2013). Although Hortega introduced the idea of a mesodermal origin of microglia, a more recent study showed that microglia differentiate from yolk-sac derived myeloid precursor cells (Ginhoux et al., 2010). Utilizing fate mapping technology, Ginhoux et al. have demonstrated that microglia progenitor cells infiltrate into the brain from the yolk sac during early embryonic development and continue to migrate and mature in the early stages of post-natal brain development. Support for the yolk sac hypothesis of microglia origin has prompted scientists to investigate the mechanisms by which microglia maintain homeostatic presence in the CNS throughout the lifetime. Several studies have found that microglia are largely maintained by proliferation, while circulating peripheral monocytes only contribute to the microglia population in disease conditions (Ginhoux et al., 2010; Bruttger et al., 2015). In adulthood, microglia make up approximately 0.5–16.6% of all cells in the brain, depending on brain sub-regional variations (Mittelbronn et al., 2001). They are widely present in the entire CNS, including the brain and spinal cord.

### Microglia morphology: a correlation with functional profiles?

One particularly essential characteristic of microglia is their ability to rapidly change morphology and function in response to changes in their microenvironment (Karperien et al., 2013). Several studies have suggested that microglia morphology falls on a spectrum, ranging from amoeboid to ramified (Stence et al., 2001; Fontainhas et al., 2011). Additionally, newer studies have introduced a third morphological classification; reactive or “alternatively” activated microglia, characterized by thick retracted processes, typically directed toward a lesion or site of protein aggregation (Franco and Fernández-Suárez, 2015). Morphologically, this state falls in between amoeboid and ramified. Ramified microglia, frequently defined as homeostatic or surveying microglia, are characterized by dynamic thin processes extending out from a relatively circular-shaped soma (Kreutzberg, 1996; Fontainhas et al., 2011; Karperien et al., 2013). Surveying microglia are involved in CNS homeostasis by actively making contacts with surrounding synaptic elements. Interestingly, several studies have demonstrated that the most complex microglia appear to be seen in compromised conditions and may be subtly activated, suggesting that ramified microglia may also be slightly reactive (Hinwood et al., 2012; Karperien et al., 2013). Amoeboid microglia display the greatest level of motility, facilitated by a retraction and reduction of processes (Kreutzberg, 1996; Karperien et al., 2013). Amoeboid microglia are most commonly found during the early stages of brain development, before they undergo morphological differentiation to ramified microglia during brain maturation (Harry and Kraft, 2012; Ginhoux et al., 2013). They are also occasionally reported in inflammatory and phagocytic conditions, although their specific function in these states remains unclear (Karperien et al., 2013). Microglia morphology is strongly influenced by neurotransmitter activity. Excitatory neurotransmission significantly increases the ramification of microglia via ATP signaling (Fontainhas et al., 2011). This evidence suggests that microglial processes are highly susceptible to external cues. Although numerous studies have attempted to elucidate the correlation between amoeboid and ramified microglia and their roles in physiological and pathological conditions, the exact functional profiles of different morphological states remain widely debated.

### The function of microglia in synaptogenesis and synaptic plasticity

Microglia are known to play an important role in synaptogenesis and synaptic wiring and maintenance, which is crucial for functional brain connectivity (Ginhoux et al., 2010; Paolicelli et al., 2011). Lim et al. have recently shown that microglia-mediated release of IL-10, a pro-inflammatory molecule, led to an increase in dendritic spines (Lim et al., 2013). Concurrently, in a second study, they demonstrated that hippocampal neurons expressed IL-10 receptors during the early stages of brain maturation (Lim et al., 2013). Together, these studies suggest a causal role of microglia in synaptogenesis through microglia-mediated IL-10 signaling. Microglia maintain the ability to modulate synaptic circuits into early adulthood. A study by Parkhurst et al. revealed a microglia-dependent effect on learning-related synaptogenesis (Parkhurst et al., 2013). In addition to their role in synaptic wiring and refinement, microglia are also key modulators of synaptic plasticity. In a study investigating plasticity in the visual system, microglia-mediated synaptic remodeling in layers II/III of the V1 was shown to be activity-dependent (Tremblay et al., 2012). Additional studies have focused on other synaptic functions, such as long term potentiation (LTP), suggesting that microglia actively participate in strengthening neuronal connections through Hebbian plasticity (Penn et al., 1998). Furthermore, several studies have been published highlighting chemokine fractalkine receptor (CX3CR1) signaling and secretion of soluble molecules enhancing NMDA receptor function as key effectors in microglia-mediated modulation of synaptic plasticity (Hayashi et al., 2006; Justin et al., 2011). A study investigating the role of microglia in ocular dominance columns found that microglia modulate experience-driven plasticity in the monocular deprivation model through synaptic pruning (Sipe et al., 2016). Moreover, microglia were shown to have an indirect effect on synaptic strength through upregulation of *TNF-*α (Lewitus et al., 2016). Together, these findings reinforce the role of microglia in modulating cortical plasticity throughout the lifetime.

### Pruning: microglia-mediated phagocytosis of excess, inactive or dysfunctional synapses

Microglia provide crucial supportive functions in the CNS development starting early embryonic stages and persisting into adulthood. In their steady state, microglia play important roles in synaptic maintenance by serving as phagocytic cells, pruning excess or dysfunctional synapses (Tremblay et al., 2010). These processes are controlled by three principal mechanisms: the complement system, chemokine pathway, and activity-dependent signaling.

The complement system, a part of the innate immune response, facilitates phagocytosis in response to antigens. Complement proteins are highly expressed in neurons and glia, but selectively localized to immature synapses (Stevens et al., 2007). Microglia express complement C3 receptor CR3 (aka CD11b, Itgam, and Mac-1), which recognizes activated C3 fragments tagged to excess, immature, or dysfunctional synapses, consequently initiating phagocytosis (Carroll, 2004; Gasque, 2004; Ransohoff and Perry, 2009).

Several studies have found that microglia-mediated pruning is dependent on fractalkine signaling, which promotes survival in monocytes (Landsman et al., 2009). *CX3CR1* is specific to microglia in the brain and subset of peripheral monocytes, and responds to pruning cues from surrounding neurons. Knock out (KO) of *CX3CR1* was associated with a brief reduction in microglia and subsequent deficit in synaptic pruning, which resulted in an excess of excitatory synapses, as well as an increase in spine density and PSD95 expression (Paolicelli et al., 2011). These findings suggest that disruptions in microglia-mediated synaptic pruning are sufficient to induce deficits in brain maturation, resulting in impaired functional connectivity.

Additionally, studies have shown that pruning is activity-dependent and persists into adulthood (Tremblay et al., 2010; Schafer et al., 2012). Recently, one study has found that synaptic pruning is negatively altered in disease conditions and results in microglia-mediated synaptic loss (Hong et al., 2016). This evidence confirms the crucial role microglia play in shaping neuronal circuits throughout the lifetime, in both physiological and pathological conditions.

### Microglial phagocytosis of cellular debris

**Figure 1:**
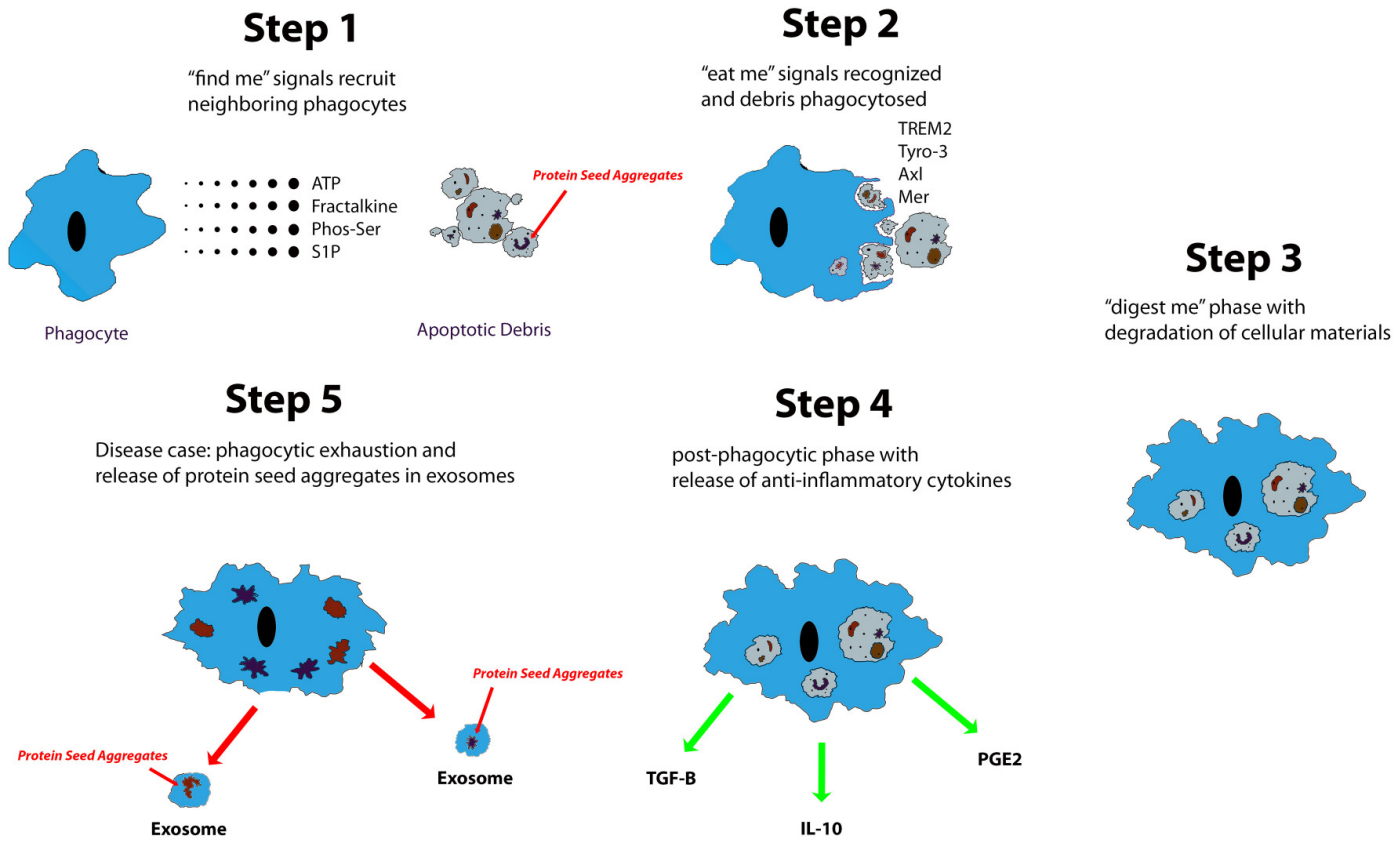
Described are five steps of apoptotic cell clearance via phagocytes. Trace chemicals and molecules associated with cell necrosis and apoptosis stimulate the chemotaxis of phagocytes up their concentration gradient to the source. From there, the phagocyte engulfs the apoptotic debris and begins degradation. Eventually, it will release anti-inflammatory and reparative signals such as TGF-β, IL-10, and PGE2. In neurodegenerative diseases of protein aggregation, protein seed aggregates are packaged into exosomes where they can be shuttled to different regions of the CNS.

Microglia are known to phagocytose biological waste and a variety of pathogens, including apoptotic bodies, cellular debris, and exogenous particles, through various well-established phagocytic pathways (Chan et al., 2001; Fu et al., 2014). This phagocytic function is crucial in both health and disease (Aderem and Underhill, 1999). Ravishadran has established a four-step model for the phagocytosis of apoptotic neurons: apoptotic cells first release “find me” signals attracting microglia, followed by the “eat me” process, mediated by specific receptors expressed by targets, the “digest me” phase, resulting in degradation of cellular materials, and finally, the post-phagocytic phase, which involves inflammatory consequences, such as cytokine and chemokine release (Ravichandran, 2010). Additionally, the fifth step, which occurs in proteopathies, has been proposed (Figure 1). Different signaling pathways are implicated in the phagocytosis of various targets (Fu et al., 2014). Extracellular nucleotides, such as ATP and UTP, are the most common “find me” signals released by apoptotic cells. UTP degradation yields UDP, which interacts directly with microglia via P2Y6 receptors (Nimmerjahn and Ravetch, 2006; Koizumi et al., 2007). Fractalkine signaling has also been shown to contribute to microglia-mediated phagocytosis of apoptotic cells (Truman et al., 2008; Noda et al., 2011; Sierra et al., 2013). “Eat me” signals are expressed by targets to initiate recognition by receptors expressed on the microglial cell surface. Toll-like receptors (TLRs) and Fc receptors are commonly implicated in microglia-mediated phagocytosis of α–synuclein (Okun et al., 2010; Hanke and Kielian, 2011; Cao et al., 2012), whereas triggering receptor expressed on myeloid cells 2 (TREM2), is known to control microglia-mediated Aβ compaction, and phagocytosis of apoptotic neurons (Piccio et al., 2007; Takahashi et al., 2007; Yuan et al., 2016). Complement and scavenger receptors are expressed by microglia and astrocytes, specifically in the pathophysiology of AD, multiple sclerosis, and amyotrophic lateral sclerosis (Husemann et al., 2002; Alarcón et al., 2005; Keren-Shaul et al., 2017). Together, these studies indicate that various types of stimulus are able to differentially trigger microglia-mediated phagocytosis via a wide range of signaling pathways. In addition to their role in phagocytosing apoptotic cells, microglia also have an established role in initiating cell death, in a process termed “phagoptosis” (Brown and Neher, 2012). Interestingly, some studies have shown that microglia-mediated “phagoptosis” can have deleterious effects in neurodegenerative diseases by triggering phagocytosis of viable neurons (Kao et al., 2011). These findings suggest that microglia-mediated phagocytosis can have both protective and deleterious effects in disease states (Fu et al., 2014).

### Microglial antigen presentation

In homeostatic physiological conditions, the blood brain barrier (BBB) prevents most peripheral infectious agents, as well as peripheral immune cells, from reaching the CNS. Consequently, the brain depends on its' own immune cells, microglia, to fulfill the roll of peripheral immune cells. As such, microglia have been shown to act as antigen presenting cells (APCs) upon activation in response to immune-related insults (Gottfried-Blackmore et al., 2009). In homeostatic conditions, major histocompatibility complex class II (MHC-II) expression is virtually non-existent in microglial populations (Ford et al., 1995). In inflammatory or neurodegenerative conditions, however, microglial MHC- II expression is quickly upregulated (Perry et al., 1987; Kreutzberg, 1996; Gottfried-Blackmore et al., 2009). One of the largest barriers to comprehending this mechanism comes from the observation that microglia are unable to travel to the lymph nodes, limiting their ability to act as APCs. Several studies have proposed that the CNS fulfills the role of a functional lymphatic system, where antigen-presenting microglia can come into contact with peripheral APCs in the meninges and choroid plexus, effectively playing an active role in T-cell mediated acquired immunity in the CNS (Louveau et al., 2015). However, recently it is speculated that antigens can be delivered to meningeal lymphatic vessels, where meningeal macrophages reside (Raper et al., 2016). There is no evidence of microglial presence in this region, suggesting their limited role for antigen presentation (Mildner et al., 2017).

### Microglial chemotaxis

Resting microglia are known to exhibit dynamic behavior, surveying their environment and making contacts with surrounding cells in order to execute a variety of functions. When activated, microglia become highly motile cells, migrating toward pathological stimuli such as debris and lesions. Chemotaxis from chemo- (chemical) and -taxis (movement), defines the ability of a cell to move in response to chemical triggers. Microglial migration is modulated by microglia-expressed receptors and gradients of different chemoattractant chemokines released by targets in inflammatory conditions (Dijkstra et al., 2004; Wang et al., 2008). The signaling pathways involved in microglia chemotaxis are highly complex. Briefly, studies have highlighted the specific roles of extracellular signal–regulated kinases (ERK1/2), protein kinase A (PKA), phospholipase A2 (PLA2), phosphoinositide 3-kinase (PI3K), and ATP/purinergic receptor signaling pathways in regulating microglial migration (Fan et al., 2017). Monocyte chemoattractant proteins (MCPs) are of particular interest in neuroinflammatory conditions. The expression of CCL2, aka MCP-1, has been associated with microglia activation in the pathogenesis of multiple sclerosis (Simpson et al., 2000) and AD (Conductier et al., 2010). Additionally, neuron-derived Fractalkine (CXCL1) stimulates microglia migration through CX3CR1 receptors (Harrison et al., 1998). Interestingly, CXCL1 is implicated in a variety of neurodegenerative disorders by acting as an anti-inflammatory agent, which may prove to be an interesting therapeutic target (Desforges et al., 2012).

### A role of microglia in neuroinflammatory conditions

A key function of microglia is their ability to respond rapidly to immune-mediated insults and physical damage in the brain. Microglia modulate the stress response to a variety of pathological triggers in CNS diseases reviewed extensively by Streit and Graeber et al. (Streit et al., 2004; Graeber et al., 2011). It is well-established that physiological responses to infection in the periphery are propagated through microglia in the CNS directly without the help of a cellular messenger (Chen et al., 2012), a response that is largely reduced after microglia depletion (Elmore et al., 2014). The propensity of the brain to propagate an inflammatory response is increased naturally due to aging (Rosczyk et al., 2008). These triggers range from autoimmune damage, such as demyelination from multiple sclerosis (Vowinckel et al., 1997; Ponomarev et al., 2005), CNS infection (Rock et al., 2004), protein aggregation (such as amyloid plaques) (Kamphuis et al., 2012), or cellular debris from neurodegeneration (Fraser et al., 2010), to physical damage from trauma or ischemia. Microglia also contain a number of purinergic receptors that respond to extracellular ATP and ADP, which are signs of possible necrosis and cellular injury (Inoue and Tsuda, 2012). Upon activation, microglial physiological function is altered, characterized by changes in morphology, coupled with upregulation of cell surface receptors and expression of chemokines and cytokines, all dependent on the triggering event (Perry and Teeling, 2013). The have been a subject of intense debate for the past few decades. Studies have demonstrated that microglia activation, independent of other cytotoxic elements, impacts synaptic function (Selkoe, 2002; Di Filippo et al., 2008) and has neurotoxic effects (von Bernhardi et al., 2015b) that correlate with neurodegeneration and decline in cognitive abilities (Cagnin et al., 2001; Kim and de Vellis, 2005). However, it is also widely believed that transient microglia activation is beneficial in neuroinflammatory conditions by promoting neuron survival (Neumann et al., 2006) and repair after brain injury (Kitamura et al., 2004) through anti-inflammatory signaling.

### How do microglia sense damage?

Microglia activation in neuroinflammatory conditions is mediated by a variety of complex signaling pathways, recently reviewed in Kaminska et al. (2016). Pathogen associated molecular patterns (PAMPs) and damage or danger -associated molecular patterns (DAMPs) activate PRRs (pattern recognition receptors) (Janeway, 1992; Kigerl et al., 2014), triggering crucial responses to immune-related insults and physical injury. PAMPs are expressed by microorganisms and play a critical role in innate immunity. DAMPs are produced by damaged cells and trigger a microglial response to brain injury. PAMPs and DAMPs have been reported to mediate microglia activation and immune response via a variety of PRRs, including Toll-like receptors (TLRs) and nucleotide oligomerization domain (NOD)-like receptors (NLRs) (Kigerl et al., 2014). TLRs are commonly activated in CNS injury and promote transcription of pro-inflammatory cytokines via activation of NF-κB and MAP kinase signaling pathways (Akira and Takeda, 2004). Lipopolysaccharide (LPS) is a PAMP of particular relevance to neurodegenerative diseases. It exerts its function through TLR4 and results in the production of several pro-inflammatory cytokines (Lu et al., 2008). *TLR4* mutations were shown to reduce microglia activation, while stimulation of TLR4 modulated cytokine expression in AD models (Jin et al., 2008). NLRs trigger the formation of large protein complexes that activate caspace-1, an essential molecule in the production of pro-inflammatory cytokines (Martinon et al., 2002; Kigerl et al., 2014). NLRP3 is expressed on microglia and participates in microglia activation in AD and several prion diseases (Halle et al., 2008; Kigerl et al., 2014). Microglia activation is also mediated by purines, such as ATP and UTP, released by apoptotic cells. These induce chemotaxis and phagocytosis via ubiquitously expressed purinergic receptors on microglial cell surfaces (Koizumi et al., 2013). Additionally, research has shown that in neuroinflammatory conditions, neurotransmitters have the capacity to mediate microglia-neuron interactions (Mead et al., 2012). Microglia express glutamate receptors, which allows them to sense neuron-released extracellular glutamate, a potent neurotoxic factor (Taylor et al., 2005). Interestingly, studies have reported that upon activation, microglia are self-producers of glutamate, which induces excito-neurotoxicity in neurons and contributes to the pathology of several neurodegenerative diseases (Takeuchi et al., 2006). This suggests that microglia are capable of autocrine signaling and supports the idea of a self-renewing neurotoxic cycle in chronic inflammatory conditions. Microglia activation is also initiated by the absence of certain signaling pathways. CD200 is expressed by neurons and interacts via CD200R expressed on microglial cell surfaces. This interaction maintains microglia in a resting, inactive state (Hoek et al., 2000). CD200 expression is downregulated with age and contributes to increased microglia activation and neuroinflammation (Lyons et al., 2007).

### Brain aging

Aging is the single most dominant risk factor for all neurodegenerative disorders, resulting in an impairment in protein production, homeostasis, chaperone-mediated folding, trafficking, stability, clearance, and autophagy. It is important to note that the CNS undergoes several changes during aging including, the shrinking of cortical areas (Salat et al., 2004; Raz et al., 2005), restricted neurogenesis (Praag et al., 2005), reduction in synaptic density, reduction in cognitive and psychomotor function in both in humans and in mice (Godbout et al., 2008; Hayashi et al., 2008), and reduction in glucose metabolism in various regions (Salmon et al., 2003; Kalpouzos et al., 2009; Hsieh, 2012). Additionally, aging also typically leads to an overall increase in the level of proinflammatory cytokines, such as IL-1β, IL-6, CD68, CD11b, and Toll-like receptors (TLRs) (Maher et al., 2004; Godbout et al., 2005), and a decrease in anti-inflammatory cytokines, such as IL-10 and IL-4 (Maher et al., 2005; Nolan et al., 2005). Furthermore, overall brain volume normally decreases by approximately 20% by the time a person turns 100 years old. It is also worth noting that the aged brain frequently has impaired vasculature, resulting in reduced oxygen and nutrient delivery to the CNS that may be exacerbated in certain brain regions (Montagne et al., 2015). Moreover, studies have suggested that blood-brain-barrier (BBB) permeability is increased by aging (Blau et al., 2012; Enciu et al., 2013), suggesting a greater susceptibility to external factors.

The natural propensity of the CNS to adopt a more inflammatory microenvironment during aging earned it the nickname “inflamm-aging” (Franceschi et al., 2000; De Martinis et al., 2005). This may be due in part to the increase in reactive oxygen species (ROS) that is evident in the aged brain. Microglia are largely responsible for the production of these species, which include lipid peroxides, superoxide anions, and hydroxyl radicals (Coatrieux et al., 2007). These molecules, in turn, lead to increased oxidative stress and elicit neurotoxic effects. Oxidative stress is known to initiate neuronal cell death *in vitro* and via a high calorie diet *in vivo* (Bros et al., 2014; Treviño et al., 2015). Conversely, some anti-inflammatory factors are known to also increase in concentration in the aged CNS. For example, TGF-β1 is a potent anti-inflammatory factor upregulated in the aged brain (Blobe et al., 2000; Tichauer et al., 2014). TGF-β1 has been shown to promote microglial phagocytosis of Aβ (Wyss-Coray et al., 2001). When considering the effect of age on various CNS responses to different stressors, aged mice, in comparison to young mice, appeared to frequently exhibit exaggerated or prolonged release of proinflammatory cytokines, worsened cognitive decline, as well as age-dependent anxiety-like behavior and sociability changes (Shoji et al., 2016).

### Microglia in aging

Microglia undergo changes in their morphology, phagocytic activity, chemotactic activity, surveying activity, and inflammatory responses that could be relevant to their involvement in disease. Research suggests age-related changes prime microglia to polarize and cause damage in response to disease-related insults. When trying to understand the true nature of pathological conditions, it is important to distinguish the changes resulting from the disease itself, vs. normal age-related variations.

Microglia undergo key morphological changes during aging. Microglial surveying processes are reported to be less dynamic, less complex, and to travel more slowly as mice age (Sierra et al., 2007; Damani et al., 2011). This suggests that responses to pathogens, aggregated proteins, or injury will be delayed in aging brains in comparison to younger mouse brains. In the aged brain, microglia appear to have enhanced proliferation in response to injury, shown in a facial nerve axotomy study in rats (Conde and Streit, 2006). Migration velocity of microglia in response to injury also appears to be affected by aging (Damani et al., 2011; Hefendehl et al., 2014). Studies have shown that in aged animals, microglia survey the environment at a lower speed (Hefendehl et al., 2014), possess thinner and fewer distal branches, and contain spheroids within the major processes (Egensperger et al., 1998; Simmons et al., 2007; Streit et al., 2009). It is possible that in these conditions, myelin fragmentation significantly contributes to the formation of these spheroid inclusions in microglia (Safaiyan et al., 2016). Furthermore, aging reduces microglia cell soma volume and results in decreased tissue distribution homogeneity. (Euler and Schuitemaker, 2012; von Bernhardi et al., 2015b). These characteristics are typically referred to as microglial dystrophy, and are considered to be a normal age-dependent phenotypic state. Recently, a new characterization termed “dark microglia” was established (Bisht et al., 2016). These microglia, identified by their extremely electron-dense soma, become more prominent with age and are especially present in disease states. It is suggested that this may represent a senescent state of microglia. Dark microglia are thought to be caused by a build-up of lipofuscin and increased mtDNA mutations (Wong, 2013).

As microglia age, they undergo many changes at the expression level that confer a heightened inflammatory response. For instance, aged microglia express more MHC-II, as well as mouse CD68 (Godbout et al., 2005; Henry et al., 2009). CD200, a membrane glycoprotein expressed on neurons, astrocytes, and oligodendrocytes, acts as a resting or pro-ramification signal for microglia, which express CD200R. Research has shown that CD200 is decreased in the human AD brain (Walker et al., 2009). CX3CL1 is a cytokine present in neurons that appears to have similar functions as CD200 in promoting microglial ramification. CX3CL1 interacts with CX3CR1, which is also widely expressed in microglia (Fuhrmann et al., 2010). Several studies have suggested that Fractalkine signaling appears to be reduced in the aged brain (Lyons et al., 2009; Bachstetter et al., 2011; Vukovic et al., 2012). Smad3, responsible for the canonical signaling pathway for TGFβ and its anti-inflammatory effects, is reduced in the aged brain (von Bernhardi et al., 2015a). Furthermore, IFN-γ, a potent activator of microglia and initiator of pro-inflammatory gene transcription (Rock et al., 2004; Klegeris et al., 2005), is increased in the aged brain. Genes conventionally known to influence microglia maturation were found to be master regulators of age-dependent changes in microglial phenotype (Wehrspaun et al., 2015). In many species, Iba-1 expression is increased in microglia due to age, often accompanied by a less ramified morphology (Streit et al., 2004), suggesting a more proliferative microglial state. It is also important to note age-related changes in astrocytes, which include an increase in glial fibrillary acidic protein (GFAP) expression, indicating a more pro-inflammatory phenotypic state (Godbout et al., 2005).

Researchers have sought to further elucidate age-associated changes by investigating the number and dynamics of existing microglia in the CNS. There does not appear to be a change in the overall number of microglia as the brain ages. However, during aging, as microglia generally become dysfunctional, they remain in the brain for longer periods of time (Mosher and Wyss-Coray, 2014). A recent study suggested that some microglia can live to be as much as 40 years old, with an average lifespan of 4.2 years and typical yearly turnover rate of 28% (Réu et al., 2017). Furthermore, the effect of peripheral monocyte infiltration on microglia phenotype must be taken into account, given that the cytokine-release profile can differ between the two (Ritzel et al., 2015). A previous study in rats has shown an age-associated increase in blood-derived monocytes, identified as CD11b+ CD45^high^ cells (Blau et al., 2012).

Research investigating the effect of long-term intraperitoneal LPS injection has suggested that some of these changes may be more prominent in certain brain regions (Hart et al., 2012). Indeed, recent studies have revealed that the effect of aging on the transcriptome of microglia is highly dependent upon location within the CNS (Grabert et al., 2016). Given that neurodegenerative diseases frequently follow a region-specific onset, it is important to consider the idea that region-specific microglia priming could contribute to this phenomenon. The fact that the cerebellum is significantly less susceptible to amyloid deposition (Johnson-Wood et al., 1997), while microglia appear to exhibit a hyper immune-alert phenotype (Grabert et al., 2016) during aging in this region, supports this notion.

During aging, microglia generally seem to exhibit an enhanced response to both CNS and peripheral insults. *In vivo*, aged mice appear to undergo an exaggerated inflammatory response to peripheral LPS injection, defined as an increased release of pro-inflammatory cytokine IL-1β (Godbout et al., 2005). Although immunoreactivity of microglia seems to be increased, age also appears to promote a senescent phenotype in microglia that reduces their functional capabilities, which appears to be accentuated *in vitro*. One study pointed out the propensity of isolated microglia to be more ramified, and exhibit a reduction in chemotaxis, phagocytosis, autophagic capacity, and overall reactivity (Caldeira et al., 2014) after aging *in vitro*. These findings were recently reproduced in the context of amyloid pathology, where microglia exhibited reduced phagocytic capabilities after 2 weeks in culture (Caldeira et al., 2017). Primary microglia isolated from 15 month old C57BL/6 were shown to have increased secretion of IL-6 in response to LPS, as well as a reduced ability to phagocytose Aβ oligomers, when compared to younger mice (Njie et al., 2012). A decrease in the ability to migrate was also noted, along with a more senescent phenotype, and less complicated inflammatory response. Furthermore, primary microglia isolated from aged mice exhibit increased release of pro-inflammatory TNF-α, IL-1β, IL-6, and IL-10 in response to challenge with LPS (Sierra et al., 2007). Aged microglia also tend to secrete more ROS, while those from young animals predominantly secrete NO (Tichauer et al., 2014). It can be difficult to distinguish whether these established changes in microglia *in vitro* are due to age or due to the effects of changing from the *in vivo* to *in vitro* environment. Nonetheless, these findings present strong evidence that aged microglia are more readily primed for activation, may be easily triggered by pathological elements in neurodegenerative diseases.

### Microglial polarization

Microglia activation *in vitro* is often classified into two categories, M1 pro-inflammatory classical activation and M2 anti-inflammatory alternative activation (Colton, 2009). This bipolar model has evolved and is now understood to represent a spectrum, where activation status can fall anywhere between M1 and M2 (Mantovani et al., 2005; Martinez and Gordon, 2014). Pro-inflammatory molecules, such as IFN-γ, TNFα, and LPS, induce the M1 classical activation of microglia (Delgado and Ganea, 2003; Martinez and Gordon, 2014). Microglia in the pro-inflammatory state secrete a variety of inflammatory cytokines, including TNF, IL-6, IL-12, IL-23, IL-1β, as well as other cytotoxic molecules, such as ROS and NO, all of which promote neurotoxicity and reinforce the inflammatory response (Delgado and Ganea, 2003; Cherry et al., 2014). Oxidative stress is implicated in nearly all neurodegenerative disorders (Gandhi and Abramov, 2012). Studies have suggested that the accumulation of reactive oxidative species results in neuronal damage and triggers apoptosis (Gilgun-Sherki et al., 2001). LPS has been show to mediate activation-induced production and secretion of ROS and reactive nitrogen species (RNS) in microglia (Dimayuga et al., 2007). Using a co-culture system of microglia and fetal neuronal cells, Chao and collaborators revealed that both LPS and IFN-γ stimulation resulted in the production of NO, which induced neurotoxicity in neuronal cells. The production of ROS and NOS was later shown to be dependent on nicotinamide adenine dinucleotide phosphate (NADPH) oxidase activity in microglia (Qin et al., 2004; Block and Hong, 2007). *In vivo*, mice deficient of NADPH oxidase were demonstrated to experience reduced nigrostriatal degeneration in response to systemic LPS injection (Qin et al., 2013), suggesting that microglia are capable of being modulated to prevent harmful activation.

The M1 pro-inflammatory profile of microglia is counter to the M2, anti-inflammatory activation state. Stein and collaborators first reported the ability of microglia to adopt anti-inflammatory properties upon stimulation by IL-4 (Stein et al., 1992). Microglia activation by IL-4 has been shown to upregulate IGF-1 production, leading to neuroprotective and regenerative effects (Butovsky et al., 2006). Additionally, other Th2-associated cytokines, such as, IL-10 and TGFα, as well as glucocorticoids, have been reported to promote M2 activation in microglia (Goerdt et al., 1999). M2 microglia have further been subdivided into three functional subclasses: M2a, M2b, and M2c (Mantovani et al., 2004; Chhor et al., 2013). M2a is induced in microglia via IL-13 and IL-4 signaling and is primarily responsible for Arg-1 production, a molecule known to participate in collagen formation facilitating tissue repair (Chhor et al., 2013). Arg1 was also recently shown to increase uptake of Aβ (Cherry et al., 2015). Microglia M2b phenotype is triggered by TLR agonists. Interestingly, the M2b subtype is capable of producing both pro- and anti-inflammatory cytokines (Bell-Temin et al., 2015). The M2c subtype, induced by IL-10, TGFα, and glucocorticoids, have two key functions following brain injury; termination of the pro-inflammatory immune response (Bell-Temin et al., 2015) and repair and regeneration after brain injury (Mantovani et al., 2004). A more recent study validated microglia's regenerative role in disease states by showing that TGFα derived from M2 microglia encourages proliferation and maturation of neural precursor cells in tissue damaged from ischemic stroke (Choi et al., 2017).

The M1/M2 classification scheme contributes to a basic understanding of well-defined microglia-mediated immunological responses *in vitro*. Several experts, however, have questioned its comprehensiveness in describing *in vivo* processes and its validity in disease states (Butovsky et al., 2014; Martinez and Gordon, 2014; Ransohoff, 2016). Microglia are thought to be particularly hard to research, given that their gene expression profiles can change fairly dramatically when taken from the CNS and placed into the *in vitro* environment (Gosselin et al., 2017). Particularly, there appears to be an increase in the expression of genes associated with inflammation and stress. SORL1, the receptor for APOE protein whose deficiency has been noted in AD patients (Scherzer et al., 2004), appears to be under-expressed in the *in vitro* environment (Gosselin et al., 2017). Isolation of primary microglia is a tenuous and complicated process. Immunohistochemistry in microglia is also noted amongst researchers to be challenging, due to issues with granular staining and autofluorescence (Koellhoffer et al., 2017). Microglia are identified by a variety of markers to distinguish them from other glial cells and neurons. Unfortunately, they share a number of these markers with peripheral macrophages, making them hard to distinguish. Recent research suggests a third, new classification of microglia, may better reflect their *in vivo* phenotypes, specifically in disease states.

### Microglia in neurodegeneration

Beyond normal aging, microglia in neurodegenerative conditions experience a specific change in phenotypic state, which researchers have struggled to characterize. Given that there are clear distinctions between microglia that promote neurogenesis and reverse atrophy, and those that release ROS and pro-inflammatory cytokines, understanding the phenotypic state responsible for mediating neuroinflammatory damage is of paramount importance. Potential therapeutic interventions should target the specific deleterious activities of harmful microglia, while leaving beneficial neuroprotective mechanisms unhindered.

Microglia activation is a necessary and beneficial function in response to acute neuro-inflammatory events and aids in sustaining brain homeostasis. Chronic activation, however, can occur from excessive neuronal or immune-related damage in various CNS diseases (Polazzi and Monti, 2010). This can lead to the sustained release of pro-inflammatory molecules and harmful production of ROS which results in detrimental effects. Moderate increases in these cytokines are generally considered a normal part of aging. However, large increases, as observed in AD, lead to excessive neurotoxicity (Giunta et al., 2008; Glass et al., 2010). In turn, increased neurotoxicity triggers additional microglial activation, initiating a harmful loop of inflammation and neuronal damage termed “reactive microgliosis” (Streit et al., 1999). Microglia have been shown to be activated in nearly all neurological disorders (Neumann et al., 2009). Signs of microglia activation have been reported in autoimmune diseases, such as multiple sclerosis (Goldmann and Prinz, 2013; Luo et al., 2017), prion diseases, such as Creutzfeldt-Jakob Disease (CJD) (Aguzzi and Zhu, 2017), neurodegenerative diseases, such as Parkinson's Disease (PD) and AD, as well as traumatic brain injury (TBI) and ischemia (Jassam et al., 2017; Liu et al., 2017). Their implication in the pathophysiology of such a wide variety of neurological disorders has made them an interesting target for potential therapeutic approaches.

With regards to AD, some observations can be confusing. As mentioned in the previous section, aged microglia frequently become “dystrophic” and are highly immunoreactive. However, studies have reported reduced phagocytosis of Aβ in older AD mice (Floden and Combs, 2011), which may be caused by decreased expression of CD36, an Aβ interacting protein. In AD patients, as well as mouse models of AD, Smad3 signaling appears to be reduced (Tesseur et al., 2006; Ueberham et al., 2006), likely resulting in the pathological activation of microglia. Overall microglia number in both Alzheimer's patients and AD mouse models is increased and correlates with disease severity (Olmos-Alonso et al., 2016). This suggests that disease pathology promotes microglia proliferation. Microglia behavior in AD may also depend heavily upon the stage of the disease. One study reported that Aβ fibrils enhance microglia phagocytosis, while Aβ oligomers attenuate phagocytosis (Pan et al., 2011). This finding suggests that microglia presence is more significant after considerable protein deposition.

**Figure 2:**
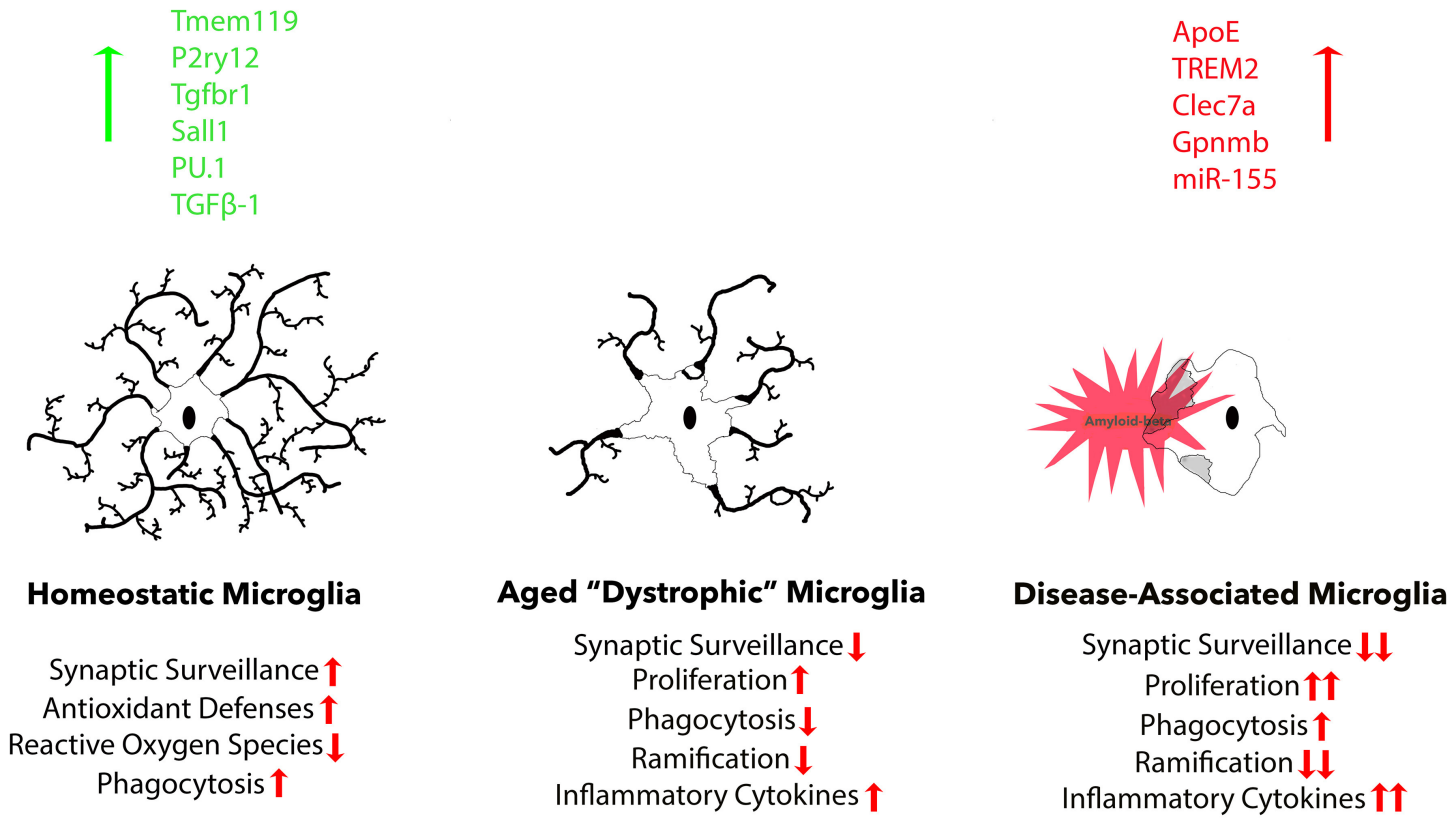
Three common microglia phenotypes are described. Homeostatic microglia are found in the adult brain under non-infectious, non-diseased, and non-aged conditions, exhibiting robust expression of homeostatic microglial markers: Tmem119, P2ry12, Tgfbr1, and transcription factor Sall1. During normal aging, homeostatic markers gradually decline, resulting in reduced functional aspects, including proliferation, phagocytosis, ramification, and cytokine secretion. Finally, there is a distinct microglia phenotype that is associated with neurodegeneration that possesses a more exacerbated dystrophic phenotype, but is specifically associated with plaques and dystrophic neurites that cause neurodegeneration.

A recent study expanded current microglia classification beyond the typical M1/M2 scheme. Researchers in this study named this third category of microglia, “disease-associated” microglia (MGnD), using a gene expression profile revealed through K-means clustering. These microglia revealed a reduction in the expression of 68 homeostatic microglial genes and upregulation of 28 inflammatory molecules (Krasemann et al., 2017). A large portion of these responses were eliminated due to microglia-specific KO of *APOE*, suggesting that APOE potently induces phenotypic changes in disease-associated microglia and is up-regulated in the presence of plaques (Krasemann et al., 2017). MGnD microglia also exhibited a significant increase in miR-155 expression. MiR-155, a significantly up-regulated microRNA in microglia after challenge with an insult, is largely responsible for the release of pro-inflammatory cytokines IL-6, IL-1β, NOS2, and TNFα (Woodbury et al., 2015). Beyond microglia interaction, APOE is important for maintaining hippocampal neurogenesis and suppressing astrogenesis in mice, both of which are reduced via APOE4 mutation (Li et al., 2009). *TREM2* KO was shown to have very similar effects on the gene expression profile as the *APOE* KO, suggesting these two molecules work concurrently to determine microglia phenotype. Another large study utilizing single-cell RNA-seq to identify unique microglia subpopulations present in 5XFAD mice found gene expression profiles associated with increase in APOE, TREM2, and Cst7 expression, amongst others, in disease-associated microglia, referred to as “DAM.” These same microglia exhibited decreased expression of homeostatic genes *P2RY12* and *CX3CR1* (Keren-Shaul et al., 2017). Lpl was selected as a consistent marker for the disease associated microglia subtype, and found to be present on phagocytic plaque-associated microglia positive for Thioflavin-S. The expression of genes known to influence microglia maturation and ramification such as *RUNX1, SALL 1, TAL1*, and *IRF8* is also affected by AD pathology (Olmos-Alonso et al., 2016). It is important to recognize the differences between MGnD and classically activated M1 or M2 microglia (Figure 2). MGnD are a result of chronic exposure to disease pathology and can be distinguished from M1 microglia by the presence APOE, TREM2, and M2-associated anti-inflammatory markers such as arginase 1 (Arg1) and chitinase-3-like protein (Ym1), as well as the absence of homeostatic transcription factor Egr1 (Krasemann et al., 2017). In contrast, M1 microglia activated through LPS down-regulate TREM2 expression (Kleinberger et al., 2017; Zhong et al., 2017b). Furthermore, plaque-associated microglia exhibit a hyperactive immune response to LPS injection in comparison to non-plaque-associated microglia (Yin et al., 2017), suggesting their contribution to neuroinflammation in disease states is more detrimental.

The MGnD and DAM phenotypes are largely dependent on TREM2 expression, which is up-regulated in the microglia of diseased brains and MGnD microglia specifically (Ofengeim et al., 2017; Yin et al., 2017). This evidence suggests that the APOE-TREM2 signaling cascade is responsible for the changes in gene expression profile which induce the MGnD phenotype in microglia (Keren-Shaul et al., 2017; Krasemann et al., 2017; Yin et al., 2017). Two papers recently reported conflicting results of *TREM2* KO in different mouse models of tauopathy. In hTau mice, prior to the time of expected significant neuronal loss, *TREM2* KO was reported to increase the deposition of hyperphosphorylated tau and promote a less homeostatic microglial phenotype (Bemiller et al., 2017). However, in a study investigating PS19 mice with *TREM2* KO, researchers found a reduction in brain atrophy in the absence of any changes in tauopathy or a reduction of MGnD-associated markers APOE and Cst7 (Krasemann et al., 2017; Leyns et al., 2017). Collectively, these recent studies support the idea that the APOE-TREM2 signaling pathway shifts microglia toward an MGnD phenotype, which actively contributes to the tauopathy-induced reduction of neuropil space in the entorhinal cortex of PS19 mice. During aging in the CNS, microglia become more dystrophic, suffer a reduction in functional characteristics, and begin to exhibit a gene expression signature similar to that of MGnD microglia. Although age is not required for the generation of the MGnD phenotype, aged microglia are primed to make this transition. Further research is necessary to generate a more concrete and time-dependent understanding of the APOE-TREM2 signaling complex as it relates to the MGnD phenotype.

### Therapeutics targeting protein misfolding

Several techniques exist allowing researchers to design pharmacological agents aimed at reducing the buildup of pathological Aβ. One validated method is to focus on developing inhibitors of BACE1, which is the membrane protease responsible for the beta-site cleavage of APP (Eketjäll et al., 2016). Several of these compounds have been in clinical trials in recent years, such as the Merck EPOCH trial in patients with mild to moderate AD. This trial was speculated to have failed because the drug was administered too late to substantially address the pathology. Another obstacle in clinical trials is heterogeneous patient enrollment, which introduces the possibility of including patients that do not have AD, but suffer from dementia due to a different disease. Instead of inhibiting Aβ production, some compounds are being designed to address the prion-like properties of amyloid aggregation itself. This concept uses a small molecule, which binds to nascent amyloid fibrils or aggregates, preventing nucleation or further accumulation (McLaurin et al., 2006; Habchi et al., 2016). Additionally, there are also antibodies designed to bind pathological Aβ protofibrils, a passive immunization technique in which the antigen, Aβ, is bound, leading to complement activation and phagocytosis by neighboring phagocytes (Bard et al., 2000). However, considerable evidence has suggested this pursuit may be clinically ineffective—decreasing amyloid burden in patients with an already significant degree of cognitive deficits, and hence neurodegeneration, does not appear to dampen the rate of decline (Holmes et al., 2008). This was most recently found during Phase 3 of a clinical trial for Solanezumab (Doody et al., 2014). Aducanumab, a fully human IgG isolated from cognitively normal donors that binds a conformational epitope of Aβ, is currently in Phase 3 trials (Sevigny et al., 2016). Neurofibrillary tangles (NFTs), the second pathological hallmark of AD, are another promising target for pharmacological intervention. Several classes of pharmacologic agents may be effective in preventing both the aggregation and spread of NFTs in AD. Small molecule drugs, that work by binding to tau to inhibit its aggregation, are currently being developed (Harrington et al., 2015). Additionally, several tau vaccines are also currently in development, with one entering Phase II of clinical trial (Theunis et al., 2013; Kontsekova et al., 2014).

Pharmacological modulation targeting the intracellular trafficking of APP via intervention on the enzymes involved in its cleavage may also be successful in regulating Aβ accumulation. APP is not exclusively found on the cell surface; it is most frequently localized to the trans-Golgi network (TGN) (Caporaso et al., 1994; Hartmann et al., 1997; Xu et al., 1997). After shuttling to the plasma membrane, APP is reinternalized into endosomes and, eventually, lysosomes (Haass et al., 1993a,b; Koo and Squazzo, 1994), where Aβ production primarily occurs (Huse et al., 2000, 2002). Therefore, enhanced sequestering of APP to the plasma membrane and out of the TGN is a potential therapeutic venue for AD. Currently, there are no drugs in development targeting this mechanism. TPI-287, a microtubule-stabilizing agent, is a unique drug, which influences APP intracellular trafficking, and may also facilitate kinesin-mediated axonal transport of APP (Zempel and Mandelkow, 2014).

### Strategies for targeting microglia and neuroinflammation

Given the overwhelming evidence supporting the role of microglia in the pathogenesis of neurodegenerative functions, it is of interest to discuss potential therapeutic interventions targeting the neurotoxic effects of microglia.

One strategy is to pharmacologically enhance microglial clearance of protein aggregates. Sargramostim is a synthetic form of the hematopoietic growth factor granulocyte-macrophage colony-stimulating factor (GM-CSF), which is FDA approved to increase white blood cell count after chemotherapy (Markovic et al., 2008). Evidence suggests that GM-CSF stimulates phagocytosis of Aβ via bone-marrow derived macrophages and microglia (Mitrasinovic et al., 2001), although GM-CSF may also potentially exacerbate their inflammatory response. Sargramostim was entered into two Phase 2 clinical trials to assess treatment and safety in patients with mild cognitive impairment or AD. One of these trials has since been withdrawn. Valproic acid, an anticonvulsant medication which exerts its function via inhibition of voltage-gated sodium channels and increases levels of gamma-aminobutyric acid (Löscher, 2002), can enhance microglial phagocytosis of Aβ, although it has not yet been tested in clinical trials. Additionally, vasoactive intestinal peptide, a multifunctional neuropeptide, which can enhance microglial phagocytosis, while suppressing the production of TNF-α and ROS (Song et al., 2012), could also be effective for restoring homeostatic microglial functions.

Gotz et al. previously demonstrates the acceleration of NFT formation by Aβ injection into the mouse brain (Götz et al., 2001). This finding was validated in a double transgenic mouse model. The model was created by crossing P301L tau mice with APP mice expressing the familial Swedish mutations of APP (K670N and M671L), resulting in a marked acceleration in tau pathology development (Lewis et al., 2001). The same result was reported in a different double transgenic model, created by crossed PS19 mice with APPV717F mice (Hurtado et al., 2010). The potential contribution of microglia to accelerate tau pathology in the presence of Aβ accumulation has yet to be tested. The spread of pathogenic tau protein may be facilitated by phagocytic microglia, which are activated by Aβ accumulation in the brain.

Immune cells, including microglia, are highly efficient in secreting extracellular vesicles, such as exosomes and ectosomes (Robbins and Morelli, 2014; Greening et al., 2015). Research has shown that microglia promote the spread of pathological protein aggregates through exosomes (Sarko and McKinney, 2017; Soria et al., 2017). Exosomes are extracellular vesicles that are between 30 and 150 nm in size (Zomer et al., 2010; Vlassov et al., 2012; Thompson et al., 2016) and possess a myriad of roles in the CNS and periphery (Budnik et al., 2016). They are frequently excreted by antigen-presenting cells, such as microglia (Nair-Gupta et al., 2014), but are also secreted by all other cells of the CNS (Frühbeis et al., 2012). In AD, microglia have been shown to congregate around senile plaques in order to phagocytose them, consequently secreting Aβ oligomer-containing exosomes (Rajendran et al., 2006). Exosomes isolated from AD brains also contain hyper-phosphorylated tau oligomers (Saman et al., 2012), which are interestingly found in the CSF of AD patients (Saman et al., 2012; Fiandaca et al., 2015). In ALS, exosomal TDP-43 is increased, suggesting that pathological protein-containing exosomes play a role in several neurodegenerative disorders (Iguchi et al., 2016). Exosomal spread is the proposed mechanism through which microglia enhance tau propagation in mouse models of AD (Asai et al., 2015). In this study, depletion of microglia was demonstrated to reduce the spread of AAV-induced tau pathology from the medial entorhinal cortex to the dentate gyrus.

Inhibiting microglia-mediated exosome excretion may show promising results in halting disease progression in neurodegenerative disorders. Pharmacological inhibition, via GW4869, of neutral sphingomyelinase 2 (nSMase2), which synthesizes ceramide from sphingomyelin and is critical for exosome synthesis, successfully halted the packaging of human tau into exosomes in microglia (Asai et al., 2015). In addition to preventing exosome-mediated tau propagation, GW4869 also attenuates the release of pro-inflammatory cytokines in macrophages in response to LPS, while not appearing to have potent cytotoxic affects (Essandoh et al., 2015). Increased exosome secretion was demonstrated to enhance spread of prion protein in cell culture (Guo et al., 2016), which was prevented via treatment with GW4869. Evidence for the role of exosomes in prion disease, including ALS, have been presented (Vella et al., 2007; Guo et al., 2016; Iguchi et al., 2016). However, administration of GW4869 in a mouse model of ALS expressing human mutant TDP-43^A315T^ (autosomal mutation of ALS), appeared to worsen disease phenotypes, increase levels of insoluble TDP-43, and cause cytoplasmic accumulation of TDP-43 in neurons (Iguchi et al., 2016). GW4869 was also reported to disrupt synaptic connections (Tabatadze et al., 2010), suggesting potential CNS damage with this compound. The compound impaired spatial memory, as assessed via the Morris Water maze, but not spatial recognition memory, tested in the Y maze. Chronic exposure of GW4869 was also reported to have no effect on novel object recognition (Iguchi et al., 2016), suggesting inconclusive results. An alternative strategy is necessary for regulating exosomes synthesis and secretion.

Preventing the pathogenic change in microglial phenotype and infiltration of peripheral monocytes in neurodegenerative conditions is another promising strategy. Long-term use of non-steroidal anti-inflammatory drugs (NSAIDs) was classically recognized to reduce the risk of developing AD and PD by two meta-analysis studies (Chen et al., 2005; Vlad et al., 2008). However, this was not fully reproduced in clinical trials (Vlad et al., 2008; Imbimbo et al., 2010; Breitner et al., 2011).

Cumulatively, these studies have led scientists to question whether microglia are insufficient for clearing proteinopathies, or if they engage in a mechanism that progresses the pathology. This question is complex and must incorporate the findings of cutting-edge studies, which have used the depletion of microglia via CSF1R inhibitors (PLX3397 and PLX5622 among others), to assess the role of microglia in proteinopathies and disease pathogenesis. These compounds are able to deplete virtually all microglia in the CNS after as little as 1 week of oral administration without significant side effects (Elmore et al., 2014; Dagher et al., 2015; Olmos-Alonso et al., 2016). No abnormal behaviors were observed in wild type mice, even after two months of treatment with PLX3397, as evaluated by contextual fear conditioning, elevated plus maze, open field, and Barnes maze (Elmore et al., 2014). Microglia depletion appeared to block disease-mediated increases in exploratory activity of mice, suggesting this may have a beneficial effect on cognition without significantly affecting amyloid burden (Grathwohl et al., 2009; Dagher et al., 2015; Olmos-Alonso et al., 2016; Spangenberg et al., 2016). CSF1R inhibitors are currently on clinical trials for oncology and joint neoplasm indications, but not for AD or other neurodegenerative disorders. No apparent side effects were detected in treated groups. Cognitive evaluations of patients in these trials will provide us with better information on the effects of microglia have on mental capacity.

Interestingly, replacement of microglia was tested as a means of protecting against excitotoxic injury with success (Vinet et al., 2012). In this study, microglia-deprived hippocampal slice cultures where infiltrated by exogenous microglia. Given that ROS production in microglia is deleterious, one therapeutic strategy may be to inhibit NOX (Altenhöfer et al., 2015). NADPH oxidase is largely responsible for ROS production in microglia in response to LPS (Qin et al., 2004). Furthermore, NOX activity was found to correlate with cognitive impairment in AD, while KO of *NOX* was associated with a reduction in cognitive impairment (Bordt and Polster, 2014). Studies have reported that treatment with the NOX inhibitor apocymin leads to a reduction in amyloid burden, reduced overall microglia number, and reduced cerebral amyloid angiopathy in an AD mouse model (Han et al., 2015). In addition, delivery of adeno-associated virus (AAV) containing CD200, a glycoprotein involved in maintaining a quiescent state in microglia, into the hippocampus of 6-month old Tg2576 APP mice resulted in increased neurogenesis and reduced amyloidosis (Varnum et al., 2015). Microglia were also treated with CD200 *in vitro*, which enhanced their phagocytosis and promoted an M2-like phenotype. These studies support the notion that aged microglia are largely ineffective in the brain and are incapable of significantly reducing amyloid burden. Furthermore, their inflammatory presence serves only to exacerbate cytotoxicity of neurons, leading to increased neuronal loss and cognitive impairment. More thorough assessment of these findings may be necessary, but ample evidence exists to suggest that microglia presence in the CNS is detrimental in the context of neurodegenerative disease.

This notion should be considered when interpreting AD research assessing microglial dysfunction, such as studies examining the effect of TREM2 disruption in animal models of amyloidosis. Results of these studies were controversial, with some stipulating that TREM2 in microglia increase phagocytosis and clearance of Aβ (Wang et al., 2015; Xiang et al., 2016; Yuan et al., 2016), while others have suggested that disruption of TREM2 reduces amyloid plaque load (Ulrich et al., 2014; Jay et al., 2015; Krasemann et al., 2017). These differences could be caused by the different functional role of TREM2 at the various stages of Aβ accumulation in the brain. TREM2 is up-regulated in the brains of AD patients and several mutations have been identified risk factors for late-onset AD (Frischmeyer-Guerrerio et al., 2013). TREM2 expression is increased in plaque-associated microglia (Guerreiro et al., 2013) and in particular, in the processes of microglia which are in direct contact with plaques (Yuan et al., 2016). TREM2 disruption has also been shown to reduce the overall amount of plaque-associated microglia (Krasemann et al., 2017). These were unexpected results since phagocytosis of not only Aβ, but a number of other molecules recognized by phagocytes, is dependent on TREM2 (Kleinberger et al., 2014; Xiang et al., 2016; Yuan et al., 2016; Varnum et al., 2017). However, it is important to note that complete depletion of microglia has repeatedly failed to influence overall amyloid deposition (Grathwohl et al., 2009; Dagher et al., 2015; Olmos-Alonso et al., 2016). If *TREM2* KO confers a dysfunctional microglia phenotype, which results in increased amyloid burden, then this effect is likely to be caused by the functional activity of microglia (Krasemann et al., 2017). One possibility is the anti-inflammatory effect of TREM2 signaling. Expression of pro-inflammatory cytokines IL-1β, IL-6, and TNF-α in response to challenge with Aβ42 was shown to be drastically increased due to knockdown with DAP12/TYROBP and synergized with *TREM2* KO (Zhong et al., 2017a). This also supports the notion that Aβ42 is a ligand for TREM2, although one study reported that the TREM2-TYROBP association is not affected by Aβ presence in cell culture. However, it has also been shown that TREM2 is down-regulated in primary microglia cultures in response to Aβ42 (Zheng et al., 2016), which was prevented with treatment with a JNK inhibitor (Zhong et al., 2017a). JNK inhibitors are proposed as a possible therapeutic target for AD (Yarza et al., 2015) and have entered clinical trials for a number of indications, but have no approvals to date. Nonetheless, research has repeatedly suggested that TREM2 is upregulated in disease-associated microglia (Frank et al., 2008; Frischmeyer-Guerrerio et al., 2013; Krasemann et al., 2017). These findings support the notion that dysfunctional microglia in disease contribute to a cytotoxic environment through the release of pro-inflammatory cytokines and ROS. The connection between APOE, TREM2, and microglia will continue to be investigated.

The contribution of circulating peripheral monocytes to the microglia population in the CNS has been thoroughly investigated and shown to function through parabiotic mechanisms. In these experiments, the blood streams of two mice are connected, where one mouse contains a reporter in circulating monocytes that allows them to be distinguished from those of the partner. One study found low levels of monocyte infiltration after full body irradiation in mice (Hess et al., 2004), while another study attempted the same experiment and did not find evidence of any CNS penetration (Ajami et al., 2007). Two mouse models of microgliosis, facial nerve axotomy and mSOD, did not provide any evidence of CNS penetration either. Reports were also released with evidence suggesting that bone-marrow transplanted myeloid cells can penetrate the CNS mainly when the BBB is compromised (Eglitis and Mezey, 1997; Brazelton et al., 2000; Ajami et al., 2007; Mildner et al., 2009). To test the effect of peripheral lymphocytes on amyloid clearance in brain, parabiosis experiments have been conducted in which the blood supply of young WT mice was connected to that of older APP mice. These experiments did not result in a reduction in amyloid burden, suggesting that immune infiltration of circulating monocytes is not a significant factor (Middeldorp et al., 2016). If circulating monocytes are unable to penetrate the BBB without significant damage to the BBB, this is unlikely to be a factor in neurodegenerative diseases, but could be applicable to other neurologic disorders with BBB impairment, such as traumatic brain injury or stroke.

## Conclusion

Microglia are an important aspect of CNS homeostasis, damage repair, proteinostasis, and proteopathy in aging. The aged brain shows changes in microglial phenotype, which are associated with changes in protein clearance, misfolding, aggregation, and spread. Proteopathy and neuronal cell loss in neurodegenerative conditions also contribute to a shift in microglial phenotype from homeostatic to pathological, which permanently leads to harmful inflammatory responses and further promotes cortical degeneration. This mechanism provides us with novel therapeutic targets for AD and related proteopathies.

## Author contributions

KC and AV contributed equally to the manuscript. TI wrote and edited the manuscript. All authors listed have approved this work for publication.

### Conflict of interest statement

The authors declare that the research was conducted in the absence of any commercial or financial relationships that could be construed as a potential conflict of interest.
